# Clinical Guidelines for Management of Bone Health in Rett Syndrome Based on Expert Consensus and Available Evidence

**DOI:** 10.1371/journal.pone.0146824

**Published:** 2016-02-05

**Authors:** Amanda Jefferson, Helen Leonard, Aris Siafarikas, Helen Woodhead, Sue Fyfe, Leanne M. Ward, Craig Munns, Kathleen Motil, Daniel Tarquinio, Jay R. Shapiro, Torkel Brismar, Bruria Ben-Zeev, Anne-Marie Bisgaard, Giangennaro Coppola, Carolyn Ellaway, Michael Freilinger, Suzanne Geerts, Peter Humphreys, Mary Jones, Jane Lane, Gunilla Larsson, Meir Lotan, Alan Percy, Mercedes Pineda, Steven Skinner, Birgit Syhler, Sue Thompson, Batia Weiss, Ingegerd Witt Engerström, Jenny Downs

**Affiliations:** 1 School of Biomedical Sciences, Curtin Health Innovation Research Institute-Biosciences, Curtin University, Perth, Western Australia, Australia; 2 Telethon Kids Institute, Centre for Child Health Research, The University of Western Australia, West Perth, Western Australia, Australia; 3 Department of Endocrinology and Diabetes, Princess Margaret Children’s Hospital, West Perth, Western Australia, Australia; 4 Department of Paediatric Endocrinology, Sydney Children’s Hospital, Randwick, New South Wales, Australia; 5 Faculty of Health Sciences, Curtin University, Perth, Western Australia, Australia; 6 Department of Pediatrics, Faculty of Medicine, University of Ottawa, Ottawa, Canada; 7 Division of Endocrinology and Metabolism, Children’s Hospital of Eastern Ontario, Ottawa, Canada; 8 Institute of Endocrinology and Diabetes, The Children’s Hospital at Westmead, Sydney, New South Wales, Australia; 9 Department of Pediatrics, USDA/ARS Children’s Nutrition Research Center, Baylor College of Medicine, Houston, Texas, United States of America; 10 Section of Gastroenterology, Hepatology and Nutrition, Texas Children’s Hospital, Houston, Texas, United States of America; 11 Children’s Healthcare of Atlanta, Emory University, Atlanta, Georgia, United States of America; 12 Bone and Osteogenesis Imperfecta Department, Kennedy Krieger Institute, Baltimore, Maryland, United States of America; 13 Department of Clinical Science, Intervention and Technology, Karolinska Institutet, Solna, Stockholm, Sweden; 14 Pediatric Neurology Unit, Edmond & Lily Safra Children’s Hospital, Chaim Sheba Medical Center, Tel Hashomer, Israel; 15 Center for Rett Syndrome, Copenhagen, Denmark; 16 Department of Clinical Genetics, Rigshospitalet, Copenhagen, Denmark; 17 Clinic of Child and Adolescent Neuropsychiatry, Department of Medicine and Surgery, University of Salerno, Salerno, Italy; 18 Western Sydney Genetics Program, The Children’s Hospital at Westmead, Sydney, New South Wales, Australia; 19 Disciplines of Paediatrics and Child Health and Genetic Medicine, University of Sydney, Sydney, New South Wales, Australia; 20 Department of Pediatrics and Adolescent Medicine, Medical University of Vienna, Vienna, Austria; 21 Civitan International Research Centre, University of Alabama at Birmingham, Birmingham, Alabama, United States of America; 22 Division of Neurology, Children’s Hospital of Eastern Ontario, Ottawa, Ontario, Canada; 23 Katie’s Clinic for Rett Syndrome and Related Disorders, UCSF Benioff Children’s Hospital, Oakland, California, United States of America; 24 Swedish National Rett Centre, Frösön, Sweden; 25 Department of Community Medicine and Rehabilitation, Physiotherapy, Umeå University, Frösön, Sweden; 26 Department of Physiotherapy, Ariel University, Ariel, Israel; 27 Department of Pediatrics and Neurology, University of Alabama at Birmingham, Birmingham, Alabama, United States of America; 28 Fundació Hospital Sant Joan de Déu, Barcelona, Spain; 29 Centre for Biomedical Research on Rare Diseases, Instituto de Salud Carlos III, Barcelona, Spain; 30 Greenwood Genetic Center, Greenwood, South Carolina, United States of America; 31 Genetic Metabolic Disorders Service, The Children’s Hospital at Westmead, Sydney, New South Wales, Australia; 32 Division of Pediatric Gastroenterology and Nutrition, Edmond & Lily Safra Children’s Hospital, Tel Hashomer, Israel; 33 Chaim Sheba Medical Center, Tel Hashomer, Israel; 34 Neuropediatrics, Swedish National Rett Center, Frösön, Sweden; 35 School of Physiotherapy and Exercise Science, Curtin University, Perth, Western Australia, Australia; Institute of Genetics and Biophysics, ITALY

## Abstract

**Objectives:**

We developed clinical guidelines for the management of bone health in Rett syndrome through evidence review and the consensus of an expert panel of clinicians.

**Methods:**

An initial guidelines draft was created which included statements based upon literature review and 11 open-ended questions where literature was lacking. The international expert panel reviewed the draft online using a 2-stage Delphi process to reach consensus agreement. Items describe the clinical assessment of bone health, bone mineral density assessment and technique, and pharmacological and non-pharmacological interventions.

**Results:**

Agreement was reached on 39 statements which were formulated from 41 statements and 11 questions. When assessing bone health in Rett syndrome a comprehensive assessment of fracture history, mutation type, prescribed medication, pubertal development, mobility level, dietary intake and biochemical bone markers is recommended. A baseline densitometry assessment should be performed with accommodations made for size, with the frequency of surveillance determined according to individual risk. Lateral spine x-rays are also suggested. Increasing physical activity and initiating calcium and vitamin D supplementation when low are the first approaches to optimizing bone health in Rett syndrome. If individuals with Rett syndrome meet the ISCD criterion for osteoporosis in children, the use of bisphosphonates is recommended.

**Conclusion:**

A clinically significant history of fracture in combination with low bone densitometry findings is necessary for a diagnosis of osteoporosis. These evidence and consensus-based guidelines have the potential to improve bone health in those with Rett syndrome, reduce the frequency of fractures, and stimulate further research that aims to ameliorate the impacts of this serious comorbidity.

## Introduction

Rett syndrome (RTT), although considered rare, is one of the most common causes of intellectual disability in females with an incidence of 1 in 8,500 by the age of 15 years [1]. Most individuals with RTT express a mutation in the *MECP2* gene, which either activates or represses neural transcription when it binds to methylated cytosines in DNA [2,3]. However the severity of the disorder varies depending on the type of *MECP2* mutation [4–6] and the pattern of X-chromosome inactivation [7]. Clinical outcomes for this syndrome are complex, with varying degrees of autonomic dysfunction [8], motor impairments influencing mobility and oromotor control [5,9–11], epilepsy [12,13] and poor growth [14,15]. A high proportion also develop skeletal abnormalities such as scoliosis [16,17], low bone density and mass and a high frequency of fractures [18]. As almost 60% of those with RTT will live beyond 37 years, physicians must begin to address these chronic health issues early in life [19].

Studies assessing bone mineral content (BMC, g) and areal bone mineral density (aBMD, g/cm^2^) in RTT have shown that these parameters were lower compared to the gender matched control groups [18,20–22]. Bone mineral content and aBMD z-scores for age and height tended to become more negative with age [18,21–23], however individuals as young as three and four years of age had low BMC and aBMD values in the lumbar spine [18,23], total body and femoral neck [18].

Fractures are also a common occurrence in RTT. The fracture incidence in an Australian RTT population was shown to be 43.3 per 1000 person years, which equates to a rate nearly four times that of the general population [24] within whom fractures are often associated with sport and playing activities [25]. Fractures can occur spontaneously, with trivial trauma or a fall, and occur predominantly in the long bones of the upper and lower limbs [24,26]. Fractures have also been closely linked with mobility levels and ability to bear weight, and in a Danish study there were significantly more non-ambulant patients who had fractured compared to the ambulant non-fractured healthy control group [26]. To date the prevalence and incidence of vertebral fractures in RTT remains unknown. As vertebral fractures, often considered a manifestation of osteoporosis, may be asymptomatic, they may escape medical attention [27].

Our Australian study, which assessed the BMC and aBMD in the lumbar spine, femoral neck and total body using dual energy x-ray absorptiometry, found that subjects who were unable to walk or wheelchair dependent had lower mean height calculated BMC and aBMD z-score values compared to those who were ambulant [18]. The z-scores were predicted for height and sex, as these were more appropriate to use than comparisons with normal values by age, given the generally small stature of those with RTT. In a US study (n = 49), one third of individuals who were non-ambulant had decreased bone mass in the lumbar spine [23]. Small bone size and reduced lean tissue mass have also been observed and correlated with low bone density and mass values [18,22]. Lean tissue mass has been shown to be a strong predictor of bone density in young women in the general population, particularly during adolescence [28]. Reduced lean tissue mass, often seen in those who are immobilised, decreases the physiological adaptations that occur within bone as response to muscle forces. This negatively affects bone development (modeling), leading to narrower bones with thin cortices and reduced trabecular bone mass [29,30].

Despite the high frequency of fractures and low bone mass and density identified in RTT, information about best clinical practice for the diagnosis, treatment and prevention of osteoporosis in this disorder is lacking. Due to its rarity, many clinicians may have little or no experience with RTT. This study therefore developed clinical guidelines for the management of bone health in RTT.

## Methods

This project was coordinated from the Telethon Kids Institute in Western Australia (WA). Ethics approval was obtained from Curtin University in Western Australia.

Guideline development followed the format described by the National Health and Medical Research Council, Australia (1999) and consensus was achieved using a modified Delphi technique as used in previous studies [31]. Potential expert panel members were invited to participate via email which provided information about the study and described the development process. As approved by the Curtin University ethics committee, the expert panel members provided their consent to participate in the study via email, which was then stored on a password-protected server at the Telethon Kid's Institute.

### Literature Review and Parent Perspectives

The systematic literature search performed in 2013, reviewed the following databases: Medline, ProQuest Health and Medical Complete, PsychINFO, ScienceDirect, Web of Science, The Cochrane Library and EMBASE. Online libraries reviewed were those of the World Health Organisation, Canadian Medical Association Infobase Clinical Practice Guidelines, Geneva Foundation for Medical Education and Research, the National Guidelines Clearinghouse, National Institutes for Health and Care Excellence, Scottish Intercollegiate Guidelines and the Trip search engine. Keywords used in the search were a combination of RTT with bone, bone density, osteoporosis, osteopenia, fracture, vitamin D, calcium and exercise.

In addition, Rettnet, which is an online forum provided by Rettsyndrome.org allowing the exchange of information on aspects of RTT, was searched using the key words osteoporosis, bone, bone density, fracture, calcium, broke/broken, vitamin D, movement, exercise and brittle. Online communications were sourced from June 2009 to January 2011.

### Expert Panel

Potential panel members from around the world with clinical expertise in RTT or relevant medical specialties were identified from the literature, networks of investigators, recommendation of colleagues and RettSearch. Relevant specialties included pediatrics, pediatric neurology, endocrinology, clinical genetics, radiology, gastroenterology, nursing, physiotherapy and dietetics.

### Guidelines Development Using a Modified Delphi Technique

Based on findings from the literature, referenced statements and questions were developed and divided into two sections. Part A contained sections on clinical assessment, bone density assessment and related techniques. Part B focused on non-pharmacological and pharmacological intervention strategies. Members of the Consumer Reference Group (CRG) for the Australian Rett Syndrome Study, comprising parents of females with RTT, were given the opportunity to review the draft guidelines and comment on their comprehensiveness before they were made available to expert panel members. In addition, a clinical geneticist and a pediatric endocrinologist in Australia, both of whom have extensive knowledge and experience with RTT, tested the online version of the guidelines before it was made available to panel members.

Panel members had online access to the draft guidelines document, which was developed using HTML form and PHP script, and were able to log in using a personalised username and password. They were then able to respond to each statement by rating their level of agreement using a 5-point Likert scale (strongly agree, agree, neither disagree or agree, disagree, strongly disagree). There was also the option for panel members to provide a comment in relation to each statement. Additional questions not addressed in the literature were constructed based on the content of Rettnet postings. Panel members who felt they were not qualified to respond to a particular statement or question could choose the option “not my area of expertise”. All online responses could be edited, saved, and submitted in stages. Data were stored on a secure site in a MySQL database (Sun Microsystems, Cupertino, CA) at the Telethon Kids Institute in Perth, Western Australia.

Consensus for each statement was determined where at least 70% of responses were within one response category of the median response. New statements were formed from responses to each of the questions, and statements, where consensus was not reached, were modified in accordance with panel members’ comments. Panel members were then asked to review a second guidelines draft that included the new statements formulated following first round responses. They were not given access to their previous responses or information regarding the median responses to each statement as clear consensus had been reached in round 1, negating the need for panel members to modify their responses. This is therefore a Modified Delphi process. Using the Scottish Intercollegiate Guidelines Network grading scheme [32], statements where consensus was reached were rated according to their level of evidence. Systematic reviews and randomized controlled trials were rated as level 1, level 2 included case control or cohort studies, level 3 case report or case series and level 4 statements were supported by expert opinion.

## Results

### Literature Review and Parent Perspectives

Search of seven databases and seven online libraries identified 214 citations as potentially relevant. Of these 70 articles and three sets of guidelines were retrieved and reviewed in full text. Thirty-eight articles provided statements for inclusion in the draft guidelines. One hundred and ninety-five relevant Rettnet postings were identified. These postings were used to construct questions in the draft guidelines if statements had not already raised the issues. Seven members in the Australian CRG reviewed the first guidelines draft and provided feedback. Although the CRG supported the document, they requested additional statements be included on fracture vigilance and specific recommendations regarding vitamin D and calcium supplementation. The CRG also expressed concerns regarding the effects of contraceptive use on bone health and the efficacy of bisphosphonates on bone density.

### Expert Panel Participation

Of the 62 clinicians who were contacted, 45 (72.6%) agreed to participate in the study, ten did not respond and seven declined to participate. Thirty-eight (84.4%) of the 45 who agreed to participate provided data. Eleven (29%) were paediatric neurologists, six (15.8%) adult endocrinologists, five (13.2%) clinical geneticists, four (10.5%) pediatricians, three (7.9%) physiotherapists, two (5.3%) pediatric endocrinologists, two (5.3%) dieticians, two (5.3%) gastroenterologists, one (2.6%) nurse, one (2.6%) was a pediatric orthopaedic surgeon, and one (2.6%) was a radiologist. Participant locations were as follows; 17 (44.7%) United States of America, four (10.5%) Australia, three (7.9%) from Sweden and Israel, two (5.3%) from Denmark, Italy, Canada and one (2.6%) from France, Austria, Japan, Spain and the United Kingdom.

### Initial Guidelines Draft and Redrafting Using a Modified Delphi Technique

The draft guidelines included 41 statements, 11 questions (representing all RettNet topics) and a reference list. The comments made by expert panel members were either supportive of the responses made to statements or used to construct statements for round 2. Of the 38 participants who responded to the first round, 33 (86.7%) responded to the second round which included nine new statements. Agreement was achieved for all statements in the second round, allowing these statements to form part of the final guidelines. The final document contained 39 separate statements, all of which reached consensus and are listed in Tables 1–5.

**Table 1:** Assessment of Bone Health.

Statements	Level of Evidence*	Median Response	n/N (%)^1^
All children with a clinical diagnosis of RTT should undergo genetic testing as genotype may influence the development and management of osteoporosis	2	Neither Agree or Disagree	29/31 (93.5)
Fractures in RTT can occur due to trivial trauma	2	Agree	35/35 (100)
Clinicians need to be vigilant for potential fractures	2	Strongly Agree	35/35 (100)
Measure weight and height to calculate Body Mass Index at each clinical visit	4	Strongly Agree	27/35 (77.1)
Identify all prescribed medications at each clinical visit, particularly those that can influence bone density: eg anti-epileptic medications, proton pump inhibitors, progesterone-only medications, vitamin supplements	2	Strongly Agree	36/36 (100)
Assess pubertal development using Tanner staging	2	Agree	32/32 (100)
Pubertal development may be delayed in girls or women with RTT which puts those affected at risk of low bone mineral density	2	Agree	23/29 (79.3)
Assess mobility level by asking about the following:			
The level of assistance needed for walking	2	Strongly Agree	34/35 (97.1)
The time spent walking each day	2	Agree	35/35 (100)
The distance walked each day	2	Agree	34/35 (97.1)
The amount of time standing in a standing frame if independent standing is not possible	2	Strongly Agree	33/35 (94.3)
Assess dietary intake including:			
24 hour diet recall	2	Agree	31/33 (93.9)
Recall of food high in vitamin D	2	Agree	28/33 (84.8)
Recall of food high in calcium	2	Agree	33/33 (100)
Assessment of sunlight exposure by asking about			
Frequency of use of sunscreen and sun-protection factor/protective clothing	1,2	Agree	30/34 (88.2)
The time of the day when skin (equivalent to face and arms) is exposed to direct sunlight	1,2	Agree	31/34 (91.2)
Amount of time each day that skin (equivalent to face and arms) is exposed to direct sunlight	1,2	Agree	29/34 (85.3)
First line biochemical investigations include measurement of:			
Calcium (ideally also ionised calcium)	1,3	Agree	30/33 (90.9)
25 hydroxyvitamin D (25(OH)D)	1,3	Strongly Agree	32/33 (97.0)
Magnesium	1,3	Agree	30/33 (90.9)
Phosphorus	1,3	Agree	32/33 (97.0)
Alkaline Phosphatase (ALP)	1,3	Agree	28/32 (87.5)
Albumin	1,3	Agree	30/33 (90.7)
Second line biochemical investigations include measurement of:			
Electrolytes (ideally also ionised calcium)	4	Agree	25/27 (92.6)
Urine calcium/creatinine ratio (ideally also ionised calcium)	4	Agree	25/27 (92.6)
Bone turnover markers: N-telopeptide, collagen cross-links	4	Agree	24/27 (88.9)
Parathyroid hormone (PTH) if any pathological findings	4	Agree	29/33 (87.9)

*Scottish Intercollegiate Guidelines network

^1^Numerator is the number of responses with median response or 1 category either side and denominator is the number of clinicians in the panel whose expertise were relevant to this item

**Table 2:** Bone Mineral Density Assessment.

Statements	Level of Evidence*	Median Response	n/N (%)^1^
Bone health needs to be considered early on in life and the following routine risk factors should be assessed:			
Ability to walk	2	Strongly Agree	32/33 (97.0)
Presence of either the p.R168X, p.R255X, p.R270X or p.T158M mutation	2	Strongly Agree	28/31 (90.3)
Prescribed anticonvulsant medication(s)	2	Strongly Agree	32/33 (97.0)
Oral and intramuscular progesterone medication(s)	2	Agree	31/32 (96.9)
In the presence of risk factors, a baseline bone mineral density measurement should be performed	4	Agree	32/33 (97.0)
Consider using the following techniques to assess bone health:			
Densitometry (DXA)	4	Strongly Agree	25/25 (100)
Lateral spine X-ray	4	Neither Agree or Disagree	20/25 (80.0)
Peripheral quantitative computed tomography (pQCT)	4	Neither Agree or Disagree	23/25 (92.0)
Monitor bone mineral density every 1–2 years depending on clinical presentation	4	Agree	29/34 (85.3)
If long bone was fractured, the bone mineral density should be measured in the alternate bone	4	Agree	2327 (85.2
If a vertebrae was fractured, the bone mineral density may be measured in adjacent vertebrae excluding measurement of the fractured vertebrae	4	Agree	25/27 (92.6)

*Scottish Intercollegiate Guidelines network

^1^Numerator is the number of responses with median response or 1 category either side and denominator is the number of clinicians in the panel whose expertise were relevant to this item

**Table 3:** Bone Mineral Density Assessment Technique.

Statements	Level of Evidence*	Median Response	n/N (%)^1^
Where local normative data exists, measure the bone mineral content and areal bone mineral density in the total body minus the cranial bones (headless), and the postero-anterior lumbar spine	1	Agree	17/18 (94.4)
Total hip and proximal femur bone mineral content and areal bone mineral density measurements are not considered a reliable site for measurement due to difficulties with subject positioning	1	Agree	14/17 (82.4)
Z scores should be calculated from raw values for the following:			
Age	2	Agree	22/24 (91.7)
Height	2	Agree	23/23 (100)
Bone mineral apparent density (or volumetric bone mass density) adjustment is also recommended where possible	1	Agree	21/21 (100)
The same skeletal sites should be assessed when repeating densitometry measures longitudinally	4	Agree	27/27 (100)
In individuals with spinal rods, the bone mineral content and areal bone mineral density for the lateral distal femur and the total body minus the cranial bones (headless) should be measured	4	Agree	17/17 (100)
To reduce unnecessary movement during bone mineral density scan procedures, calming techniques such as music, the presence of carers/parents, swaddling or sedation may be used	4	Agree	31/31 (100)
Where possible densitometry measurements of lean tissue mass should be assessed	2	Agree	16/17 (94.1)

*Scottish Intercollegiate Guidelines network

^1^Numerator is the number of responses with median response or 1 category either side and denominator is the number of clinicians in the panel whose expertise were relevant to this item

**Table 4:** Non-Pharmacological Intervention.

Statements	Level of Evidence*	Median Response	n/N (%)^1^
Increase physical activity in order to increase muscle strength and bone density	2	Strongly Agree	34/34 (100)
In order to increase physical activity, refer to a physiotherapist for development of an optimal physical activity plan	4	Strongly Agree	33/34 (97.0)
For those who are wheelchair bound, where possible:			)
Encourage supported standing during transferring	1	Agree	33/34 (97.0)
Use a standing frame for at least 30 minutes a day	1	Strongly Agree	28/33 (84.8)
For those who are able to walk, aim to increase the distance and/or the length of time walked each day (aiming for 2 hours per day where possible)	1	Agree	29/32 (90.6)
Where mobility is limited, targeted exercise such as body weight supported treadmill or assisted walking is recommended	2	Strongly Agree	31/32 (96.9)
If calcium intake is low, increase dietary intake of calcium rich or calcium fortified foods	1,2,3	Strongly Agree	31/32 (96.9)
If dietary calcium intake is low and difficult to increase using dietary means, prescribe calcium supplements to meet the local recommended daily intake. The current recommended dietary intake levels within Australia are: 1-3yr 500mg/day, 4-8yr 700mg/day, 9-11yr 1000mg/day, 12-13yr 1300mg/day, 14-18yr 1300mg/day, >18yr 1000mg/day of elemental calcium. When prescribing medication please verify the content of elemental calcium in the preparation	1	Strongly Agree	31/32 (96.9)
If 25 hydroxyvitamin D levels are lower than 75nmol/L:			
Use local protocols for treatment and supplementation	1	Strongly Agree	27/32 (84.4)
Re-assess 25 hydroxyvitamin D levels after 4–8 weeks, then annually	4	Agree	32/32 (100)
Advise an appropriate amount of sunlight exposure based on latitude, time of day, season and skin type	1,2	Agree	27/31 (87.1)

*Scottish Intercollegiate Guidelines network

^1^Numerator is the number of responses with median response or 1 category either side and denominator is the number of clinicians in the panel whose expertise were relevant to this item

**Table 5:** Pharmacological Intervention.

Statements	Level of Evidence*	Median Response	n/N (%)^1^
Bisphosphonates should be used if the International Society for Clinical Densitometry criteria for osteoporosis in children and adolescents are fulfilled	1,2	Agree	17/23 (73.9)
The intravenous dosage of Bisphosphonates should follow evidence-based protocols	4	Agree	16/22 (72.7)
Reassess bone mineral content and areal bone mineral density one year after Bisphosphonate therapy to decide on further therapy	4	Agree	24/26 (92.3)
If reassessment of bone mineral content and areal bone mineral density shows limited response, review the therapeutic approach	4	Agree	23/26 (88.5)
If hormonal intervention for regulation of the menstrual cycle is needed, use of Depot medroxyprogesterone acetate (DMPA) should be avoided	1,2	Agree	21/21 (100)
Although Levonorgestrel-releasing intrauterine system (LNG-IUS, Mirena) does not negatively affect bone density, communication difficulties during insertion need to be considered	4	Agree	15/15 (100)

*Scottish Intercollegiate Guidelines network

^1^Numerator is the number of responses with median response or 1 category either side and denominator is the number of clinicians in the panel whose expertise were relevant to this item

### Clinical Assessment of Bone Health, Bone Mineral Density Assessment and Technique

Twelve items included in the clinical assessment of bone health section focused on factors that influence bone density in RTT such as genotype, fracture susceptibility, mobility level, height and weight, prescribed medications, pubertal development, calcium, dietary intake of calcium and vitamin D as well as vitamin D synthesis from sunlight exposure. This section also identified biochemical markers to be measured when assessing bone status in RTT. The statement “Pubertal development may be delayed in girls or women with RTT which puts those affected at risk of low bone mineral density” was added for round 2 in response to panel members’ comments regarding the atypical pubertal development seen in RTT [33,34] (Table 1). Although all statements regarding assessment of mobility reached between 94–100% agreement, several comments were made regarding the difficulties in monitoring the duration and distance of walking. Panel members also commented on the need for a dietitian to assess dietary intake in RTT.

Table 2 includes five statements relating to bone mineral density assessment. Clinicians commented that risk factors for poor bone health need to be taken into consideration when deciding if and when bone mineral density assessments should be performed. All panel members agreed that baseline bone mineral density should be assessed by DXA. The initial guidelines draft posed a series of questions on timeframes for which scans should be performed based on specific z-score results at baseline and the presence of risk factors. In response to feedback, these questions were removed and replaced with a more generalized statement that monitoring of bone mineral density should occur every 1–2 years depending on clinical presentation, taking into account z-score results as well as previous occurrence of fractures.

Table 3 includes seven statements related to techniques of assessing bone mineral density. Agreement by the panel for weight-calculated z-scores from DXA scans was not achieved (68.2% consensus) but was achieved for age and height-calculated z-scores. Techniques recommended to reduce unwanted movements during the DXA scan included listening to music, presence of parents or caregivers, swaddling and sedation when required.

### Non-Pharmacological and Pharmacological Interventions

The nine items describing the non-pharmacological intervention strategies, shown in Table 4, involved two major avenues for improving bone health; increasing physical activity and calcium and vitamin D supplementation. Although consensus was achieved for all statements, some panel members were nevertheless uncertain of the benefits of the use of a standing frame on bone density. This statement reached 84% consensus. The statement in round 1 that related to targeted exercise for those with limited mobility was changed from recommending whole body vibration therapy to assisted walking due to lack of consensus. Subsequent to this change 97% agreement was achieved.

The six items in Table 5 address the pharmacological approaches to increasing bone density when there is a combination of low bone density and a fracture history. At the time of the study we followed the 2007 position statement by the International Society of Clinical Densitometry (ISCD) for classification of osteoporosis in children and adolescents [35]. Following the development of these guidelines the ISCD updated this position statement [36]. Whilst the group agreed that bisphosphonate therapy may be indicated, several panel members did make note of the lack of evidence for efficacy and safety of bisphosphonate use in RTT and that its mechanism of action may be different from disorders such as osteogenesis imperfecta in which its use is frequent [37]. Three statements included in round 1 that related to combined, low dose or ultra low dose contraceptives and their effects on bone were removed due to lack of consensus. Panel members felt there was either a lack of evidence to support these statements or questioned their relevance in the RTT population.

## Discussion

Reduced bone mass and density have been identified in RTT throughout childhood and into adulthood [18,21–23,38,39]. In addition bone area is reduced [18,22] and muscle mass is low [18,31]. One impetus for developing these guidelines was initially in response to concerns expressed by parents on Rettnet regarding the increased likelihood of bone fractures in RTT, as well as their questions regarding the assessment and improvement of bone density in their child. Furthermore, research indicating low bone mass and density [18] and increased fracture risk [24] in RTT also highlighted the need for these guidelines.

This project identified relevant literature relating to RTT or developmental disabilities and aspects of bone growth during childhood, adolescence and adulthood. A series of statements and questions were developed from the literature and from the listserv Rettnet. An international expert panel and a parent reference group subsequently reviewed the statements. This comprehensive document, which aims to guide clinicians on best practices for maintaining bone health and reducing fractures in RTT, was compiled using a modified Delphi technique. The panel provided expert advice for the scope of the guidelines with limited “drop out” between the first and second round review. The option “not my area of expertise” made it possible for the clinicians to only express opinion in their respective field of experience, enabling inclusion of multiple health professions in the expert panel. Using similar methods as our previously developed guidelines for management of scoliosis [17] and assessment and management of nutrition and growth [31] in RTT, the communication via email and the online review of the guidelines occurred in a timely and efficient manner.

Limitations of the study were the lack of supporting peer-reviewed literature specific to RTT for aspects of the guidelines, particularly statements relating to intervention strategies. Twenty-two of 63 statements (which includes the different parts of some statements) were formulated based on clinical experiences of the expert panel rather than peer-reviewed literature. These statements particularly related to the types of biochemical testing used to assess bone health, technique, frequency and skeletal site targeted for bone density measurements, vitamin D measurements and use of bisphosphonates to treat low bone density. There is also limited evidence on the effects of physical activity on bone mass density in RTT. Where literature specific to RTT was absent, the guidelines not only relied on the opinion of the expert panel but literature on bone health in the general population and in those for other disabilities.

Biochemical assessments were described taking into consideration the likelihood of nutritional deficits observed in RTT [11]. Radiological investigations were also outlined with techniques and analyses tailored to accommodate the decreased stature [15,40] and movement problems [5] seen in RTT. For example, the guidelines include recommendations to reduce unnecessary movements during bone density scanning procedures as individuals with RTT often display stereotyped hand movements, dystonic sustained muscle contractions or abnormal postures [41]. Lastly both pharmacological and non-pharmacological approaches to improving bone density included focusing on realistic expectations of increasing weight bearing activities, calcium and vitamin D intake. These statements were made with the proviso that the tested efficacy of this approach on preventing fractures in at–risk children with RTT is unknown although there is evidence in cerebral palsy that could be relevant. A randomised clinical trial in 26 non-ambulant children with cerebral palsy (CP) found that increasing normal standing time by 60%, with a total time between 180–675 minutes/week over the course of one year, improved vertebral volumetric trabecular bone mineral density compared to the control group with CP whose standing time was unchanged [42]. However, no change was observed in the volumetric trabecular bone mineral density of the proximal tibia [42]. A more recent study, which analysed factors associated with fracture in 536 children with CP, found that those who did not use a standing device were at a nearly four times greater risk of fracture [43]. Panel members recommended the combination of increased time standing and vitamin D and calcium supplementation.

Poor growth, continuing into adulthood, has been observed in RTT from an early age [15,40,44]. Although first observed as a deceleration of head growth, height and weight are also reduced in RTT [44], consistent with DXA findings of narrow bones for chronological age [18,22]. The cause of this growth failure is complex and often correlated with clinical severity and genotype [45]. However functional abnormalities of the digestive tract, oromotor problems and other factors that affect feeding may also lead to nutritional deficits and poor growth [31,46]. Assessing dietary intake, in particular consumption of foods high in calcium, and measuring serum levels of bone markers such as calcium and 25-hydroxyvitamin D (25(OH)D) [47], are essential steps when investigating bone health. A recent study found that dietary protein, calcium and phosphorus intakes expressed as a proportion of Dietary Reference Intakes for age and sex, showed significant positive associations with TB aBMD z-scores in RTT [48].

Typically 25(OH)D is used to assess vitamin D status, however there is some conjecture as to the optimal level to support skeletal health [49]. The clinical practice guidelines for the evaluation, treatment and prevention of vitamin D deficiency developed by the Endocrine Society in the United States (US) and our guidelines, recommend that circulation 25(OH)D levels should reach a minimum threshold of 30ng/ml (75nmol/litre) [49]. However, the committee at the Institute of Medicine in the US, suggest a lower threshold at 20ng/ml (50nmol/litre) as they felt there was little or no benefit to bone health above this value [50]. The current position statement for Australia and New Zealand and others, conclude that further research is needed to evaluate non-skeletal benefits of 25(OH)D levels >50 nmol/L /litre (30ngmL) [51,52]. As the precise level recommended in RTT is unknown, given the altered bone in this condition, it may be beneficial to aim for a higher minimal threshold, although our panel recommended adherence to local protocols. The guidelines also include the recommended dietary reference intakes for calcium based on Australian standards [53]. These serve as a guide only and may differ from a clinician’s local protocol. We therefore suggest clinicians follow their local recommended calcium reference intakes. Where dietary intake is lacking or bone marker serum levels are low, supplementation is suggested.

Growth failure also needs to be considered when assessing bone mass and density using DXA analysis as the two-dimensional measurement is influenced by size and underestimates a person’s BMC and aBMD when subjects are small for age [18,54]. The guidelines therefore recommends calculation of height formulated z-scores and volumetric bone mineral density (bone mineral apparent density–BMAD) where possible [55]. Overall DXA assessment was agreed upon by the expert panel, quantitative ultrasound (QUS) may also be considered given that it is less influenced by small stature as it measures the propagation of mechanical vibrations through tissue, rather than a two dimensional measurement. Quantitative ultrasound not only provides information on the density of peripheral bones such as the calcaneous, phalanx, tibia and fibula but their biomechanical properties such as elasticity and compressive strength. Furthermore, unlike DXA, QUS is non-invasive due to the absence of radiation exposure. Although QUS is not as widely accepted as DXA given that only peripheral bone density assessments can be made, its clinical use is increasing due in part to its cost effectiveness, portability and ease of use [56]. In addition, QUS has been shown to predict future fractures similarly to that of DXA [57]. With increasing normative data for peripheral bones [56,58] QUS may be considered when assessing bone health in RTT.

A further risk factor for reduced bone density is poor mobility. In the Australian study of 97 females with RTT, we found that those with reduced mobility had lower bone mass and density measurements in the lumbar spine, femoral neck and total body [18]. Decreased lean tissue mass, also identified in RTT, would reduce the physiological load placed on bone, thereby impairing bone development and decreasing bone strength [18,30]. The recommendation to increase mobility, particularly through time spent walking or weight bearing, reached a high level of agreement by panel members. A daily training program in four independently mobile individuals with RTT aged between 8.5–11 years of age, found that both physical fitness and functional capabilities improved [59]. Due to the variability in physical capabilities in RTT, referral to a physiotherapist for development of a physical activity plan may be helpful. With reduced mobility comes the need for parents and carergivers to increasingly handle the children during transfers. It is therefore important to ensure that carergivers are trained in transfer methods and use of equipment and that the home and school environments are safe, so as to reduce the risk of accident and fracture.

Delayed puberty is another risk factor for low bone density that needs to be considered in RTT [33]. Oestrogen plays an important part in bone formation during puberty by stimulating endocortical bone formation whilst inhibiting periosteal apposition [60]. Delays in puberty may therefore negatively affect bone development. Our investigation of pubertal trajectory in 213 females with RTT in Australia found that the median age of menarche (14 years) was slightly delayed compared to the normal population (12–13 years) [33] and a US study found that 19% of girls with RTT experience delayed menarche [34]. Additionally, being underweight or having the presence of the C-terminal or p.Arg168* *MECP2* mutations correlated with delayed adrenarche, thelarche and menarche [33]. The increased height velocity and weight gain, which typically occur during puberty, have also been shown to be absent in RTT and height has been shown to be below the normative mean as early as 17 months of age in RTT [40]. Therefore the clinical assessment of bone health should include measurement of height and weight and assessment of pubertal development using Tanner staging [61].

Furthermore, the prescription of medications to regulate the menstrual cycle should be reconsidered, in particular the use of depot medroxyprogesterone acetate (DMPA) as its use has been found to increase the risk of fracture in individuals with developmental disabilities [62]. An additional class of medications that are highlighted as a risk factor for low bone density is the anticonvulsants [62], in particular valproate, which we have shown increases fracture risk three-fold in RTT, compared to the risk associated with no or any other prescribed antiepileptic drug [63].

The ISCD 2013 position paper on “Fracture Prediction and the Definition of Osteoporosis in Children and Adolescents” has recently updated its definition of osteoporosis in the young. It is now recognized that vertebral fractures can occur in at-risk children with BMD Z-scores better than the traditional -2.0 standard deviation threshold. As such, the new position paper states that BMD Z-score criteria are no longer required to define osteoporosis in children with vertebral fractures. This underscores the importance of determining whether vertebral fractures are present in high-risk populations such as RTT. According to the current ISCD criteria in children without vertebral fractures, a clinically significant fracture history plus a low BMD ≤-2.0 standard deviation are both required to diagnose osteoporosis. Clinically significant fractures include either two or more long bone fractures by age 10 years or three or more long bone fractures at any age up to 19 years [36]. It should be noted that there is marked variability in the age and gender-matched BMD Z-scores that are generated by different normative reference databases, which may place an individual above or below the absolute score of -2.0 depending on the normative data that is used [64]. This further highlights the need to investigate for the presence of both vertebral and non-vertebral fractures and monitor the occurrence of future fractures when considering osteoporosis in children. Overall, the diagnosis of osteoporosis is now based on a more functional measure of bone health—fracture history (including vertebral fractures), rather than BMD alone [65].

If vertebral fractures are present with or without low BMD z-scores or clinical significant fractures combined with low z-scores persist, the use of bisphosphonates should be considered [36]. Albeit there is only one single case study supporting its use in RTT (Lotan et al., 2013), bisphosphonates have become the standard of care in children with moderate to severe osteogenesis imperfecta where low BMC and aBMD are accompanied by recurrent long bone or vertebral compression fractures (Glorieux, 2007). However, it is still possible that the low bone mass observed in RTT may be due to fewer bone forming cells with decreased bone modeling as opposed to increased bone remodeling (Motil et al., 2014). Since bisphosphonates increase bone density by inhibiting osteoclasts reducing resorption of existing bone (Glorieux, 2007), they may have a less positive effect on bone mass in RTT than in the general population. With growth, there is continued bone accretion, which together with reduced bone resorption, increases cortical bone thickness and trabecular bone number [37]. During the first 12 months of bisphosphonate therapy in children with secondary osteoporosis, spine BMC and aBMD tend to increase significantly more than total body values [66]. Due to the paucity of evidence in RTT, re-evaluation after one year of treatment is recommended.

Fracture represents a substantial burden on those with RTT and their caregivers [67] and can occur unrecognized, given the decreased pain sensitivity and communication issues observed in RTT [68]. One goal of developing this document is to reduce the frequency of fractures and improve bone health of those with this disorder. The authors, along with an international clinical panel, have successfully developed guidelines using consensus methods, for the assessment, treatment and management of low bone density in RTT, taking into consideration the nutritional deficits and limited mobility in this disorder as well as other risk factors. Given the rarity of this disorder, these guidelines may be used by clinicians with little or no experience managing patients with RTT. Wherever possible, statements were supported by peer-reviewed literature. However in the absence of evidence, the guidelines relied heavily on expert panel opinion. The lack of supporting literature opens the door for further research to enhance our understanding of how to improve bone density in RTT and the identification of successful treatment protocols when bone mass and density are reduced.

## Abbreviations

RTT: Rett syndrome
TB: total body
LS: lumbar spine
DXA: densitometry
aBMD: areal bone mineral density
BMC: bone mineral content
vBMD: volumetric bone mineral density
LD: large deletion
CT: carboxyl-terminal
